# Panel Associations Between Newly Dead, Healed, Recovered, and Confirmed Cases During COVID-19 Pandemic

**DOI:** 10.1007/s44197-021-00019-z

**Published:** 2021-12-11

**Authors:** Ming Guan

**Affiliations:** 1 International Issues Center, Xuchang University, No. 88 Road Bayi, Xuchang, Henan China; 2 Family Issues Center, Xuchang University, No. 88 Road Bayi, Xuchang, Henan China; 3 School of Business, Xuchang University, No. 88 Road Bayi, Xuchang, Henan China

**Keywords:** Newly dead cases, Newly recovered cases, Newly healed cases, Newly confirmed cases, Panel associations

## Abstract

**Background:**

Currently, the knowledge of associations among newly recovered cases (NR), newly healed cases (NH), newly confirmed cases (NC), and newly dead cases (ND) can help to monitor, evaluate, predict, control, and curb the spreading of coronavirus disease 2019 (COVID-19). This study aimed to explore the panel associations of ND, NH, and NR with NC.

**Methods:**

Data from China Data Lab in Harvard Dataverse with China (January 15, 2020 to January 14, 2021), the United States of America (the USA, January 21, 2020 to April 5, 2021), and the World (January 22, 2020 to March 20, 2021) had been analyzed. The main variables included in the present analysis were ND, NH, NR, and NC. Pooled regression, stacked within-transformed linear regression, quantile regression for panel data, random-effects negative binomial regression, and random-effects Poisson regression were conducted to reflect the associations of ND, NH, and NR with NC. Event study analyses were performed to explore how the key events influenced NC.

**Results:**

Descriptive analyses showed that mean value of ND/NC ratio regarding China was more than those regarding the USA and the World. The results from tentative analysis reported the significant relationships among ND, NH, NR, and NC regarding China, the USA, and the World. Panel regressions confirmed associations of ND, NH, and NR with NC regarding China, the USA, and the World. Panel event study showed that key events influenced NC regarding USA and the World more greatly than that regarding China.

**Conclusion:**

The findings in this study confirmed the panel associations of ND, NH, and NR with NC in the three datasets. The efficiencies of various control strategies of COVID-19 pandemic across the globe were compared by the regression outcomes. Future direction of research work could explore the influencing mechanisms of the panel associations.

## Introduction

Despite travel restrictions [1] and limitations [2], coronavirus disease 2019 (COVID-19) has rapidly spread across the globe as a result of multiple literature. For instance, a longitudinal analysis concluded the impact of COVID-19 could migrate between vulnerable counties [3]. Another theoretical study demonstrated that a large-scale spatial transmission of COVID-19 was caused by the relatively high per-capita rate of transmission [4]. To tackle the spread of COVID-19, a growing number of countries initiated practical strategies (in-house isolation, quarantine, and promoting general awareness about transmission routes) against further development of contagion [5]. But consequently, the situation rapidly deteriorated with increasing number of newly confirmed cases (NC) [6], especially in western countries. Especially, a certain empirical law of COVID-19 spread attracted academic attention [7]. Although NC between countries was reported [8], national gaps among newly recovered cases (NR), newly healed cases (NH), and newly dead cases (ND) were seldom documented in the current academic literature. Statistical analyses with micro and macro data of COVID-19 pandemic can help evaluate the relevant control interventions.

Till now, regarding the epidemic evolution of total COVID-19 infections, analytical methods of control efficiency of COVID-19 pandemic are limited and biased. Notably, trend forecast with publicly available micro epidemiological data has been particularly the mainstream in the field of COVID-19 control. For example, multiple studies forecast a trend of the COVID-19 spreading in China [9–11]. Moreover, the temporal dynamics of the COVID-19 epidemic were reported in the parts of the World including Huangshi city, China [12], South Korea [13], UK and Sweden [14], Pakistan [15], and Wuhan, China [16]. The survival duration including the average lag between NC and ND [17], lethal duration [18], and COVID-19 duration [19] were employed to reflect the evolution of COVID-19 pandemic. But, forecast and trend methods often considered time change and neglect the relationships among ND, NH, NR, and NC. Additionally, pure mathematics underlined prediction errors caused by large uncertainties [20]. However, those studies without regional, national, and global variables could not obtain correct and scientific findings.

To date, analytical tools in published studies were limited to reflect the associations of ND, NH, and NR with NC. For example, a substantial body of time series models and simulations employed not spatial and locational factors but temporal factors [21–26]. Several simulations reported time trend of ND, NH, NR, and NC, but provided limitations in studying locational differences [27–29]. Thus, time series studies and simulations led to partial and biased research outcomes. Even more importantly, panel associations of ND, NH, and NR with NC were not analyzed.

Furthermore, policy interventions were not considered in the current studies. From December 12, 2019 till now, a series of daily policies and regulations were released by the Chinese government, global organizations, and western countries and documented in China Data Lab [30]. With publicly available data of the COVID-19 pandemic for both the USA and Italy, a study observed that the future NC, ND, and NR of COVID-19 were reasonably predicted [31]. Thus, trend driven by policy outcomes regarding NC which indirectly assessed national struggling efforts against COVID-19 pandemic often were neglected.

The progress in COVID-19 crisis was formally characterized by ND, NH, NR, and NC. Thus, this study based on publicly available longitudinal datasets to explore panel associations of ND, NH, and NR with NC. According to the presumptions of the panel models, pooled regression, stacked within-transformed linear regression, quantile regression for panel data, random-effects negative binomial regression, and random-effects Poisson regression would be conducted to reflect the associations of interest regarding China, the USA, and the World. Subsequently, panel event study was performed to reflect the trends of NC. Consequently, the endemic control performance would be further analyzed, assessed, and compared on the basis of the empirical outcomes.

## Statistical Strategies

### Data Sources and Selection

Daily cases in China included the numbers of NH, NC, and ND at the province-level unit available from January 15, 2020 to January 14, 2021 [32]. Daily cases in the USA included the numbers of ND and NC at the state-level unit available from January 21, 2020 to April 5, 2021 [33]. Daily cases in the World (outside Antarctica, China, the USA, and MS Zaandam) included ND, NR, and NC at the country-level unit available from January 22, 2020 to March 20, 2021 [34]. The dataset of China contained information on 31 province-level units. The dataset of the USA contained information on 51 states. The dataset of the World contained information on 192 countries and regions. The geographical divisions could be found in Appendix. There was no data cleaning performed on the raw data available at Harvard dataverse.

### Front-and-Back Plots

**Fig. 1:**
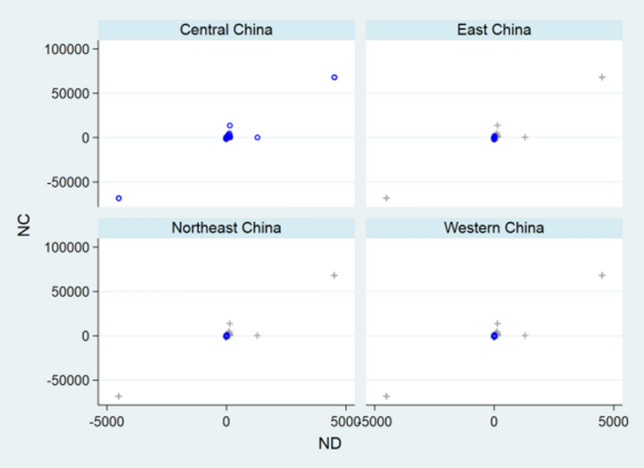
Relationship between ND and NC regarding China

**Fig. 2:**
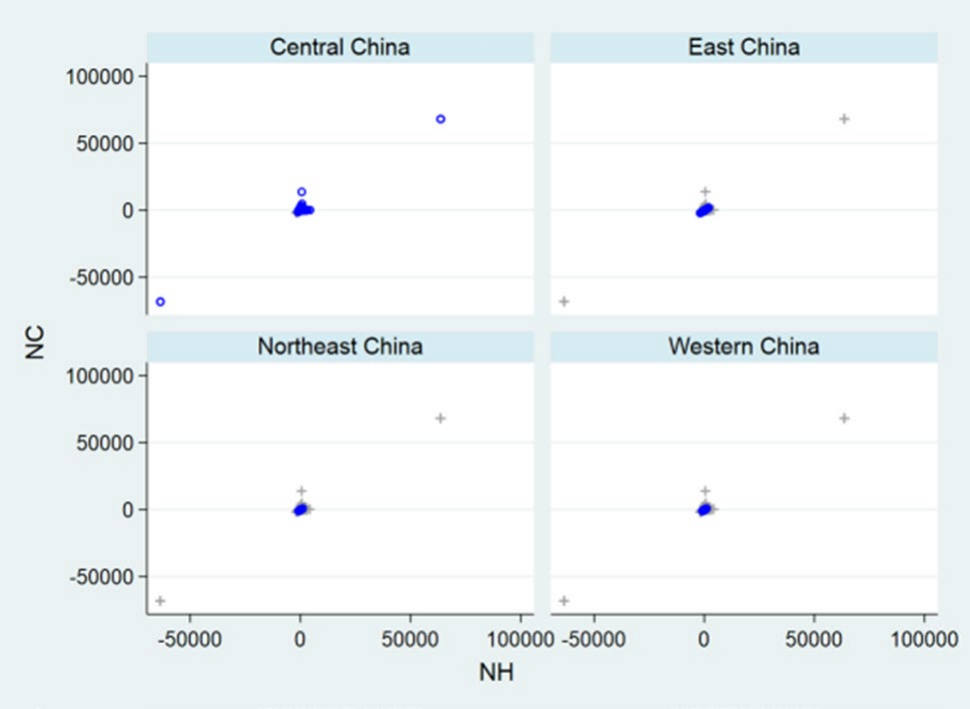
Relationship between NH and NC regarding China

**Fig. 3:**
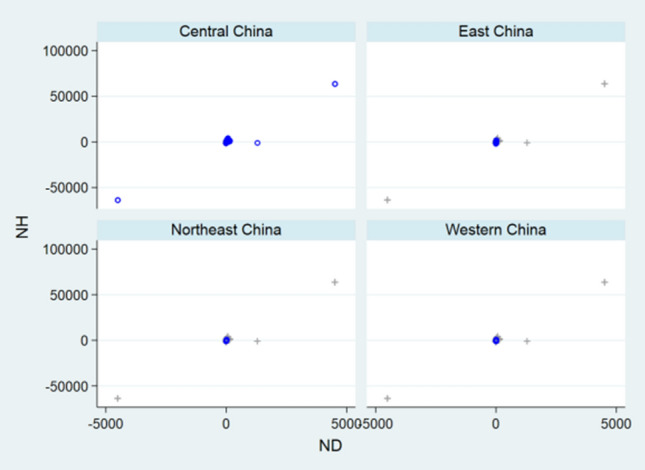
Relationship between ND and NH regarding China

**Fig. 4:**
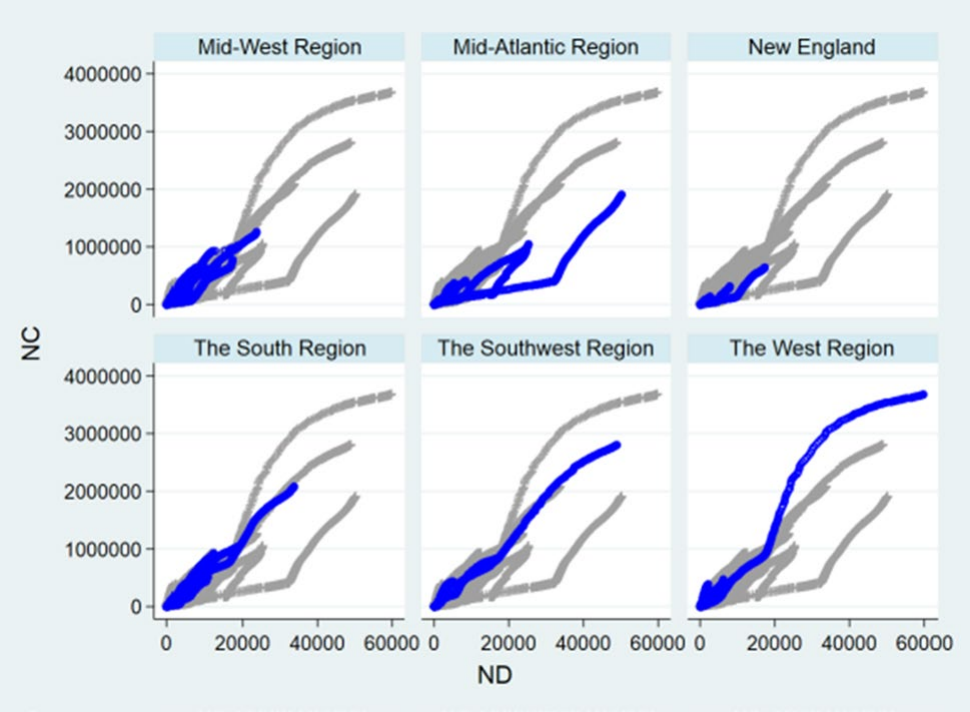
Relationship between ND and NC regarding the USA

**Fig. 5:**
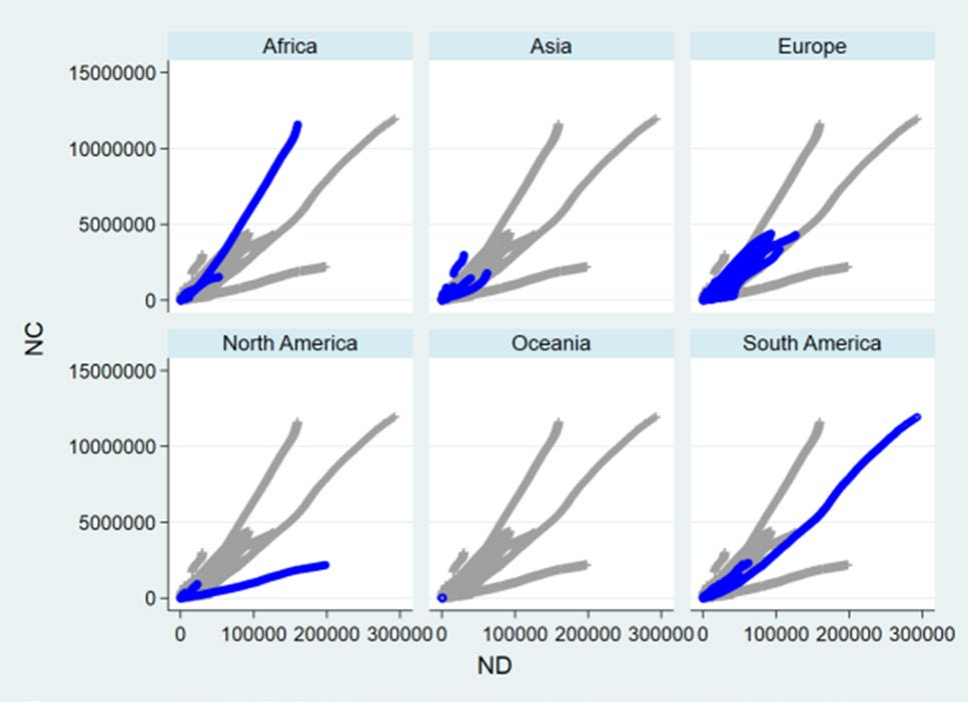
Relationship between ND and NC regarding the World

**Fig. 6:**
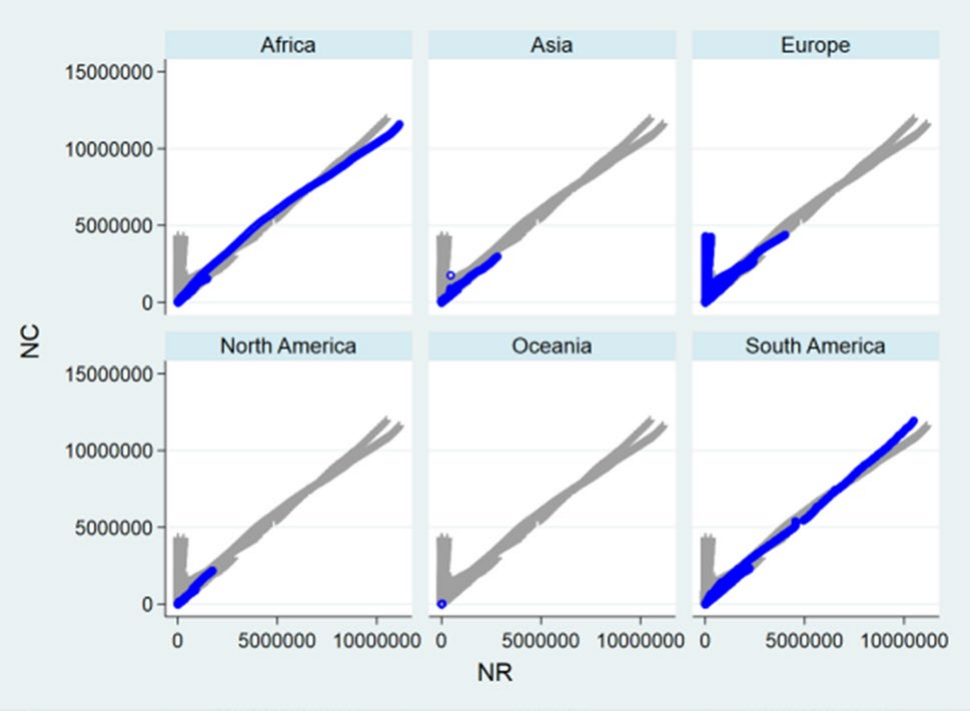
Relationship between NR and NC regarding the World

**Fig. 7:**
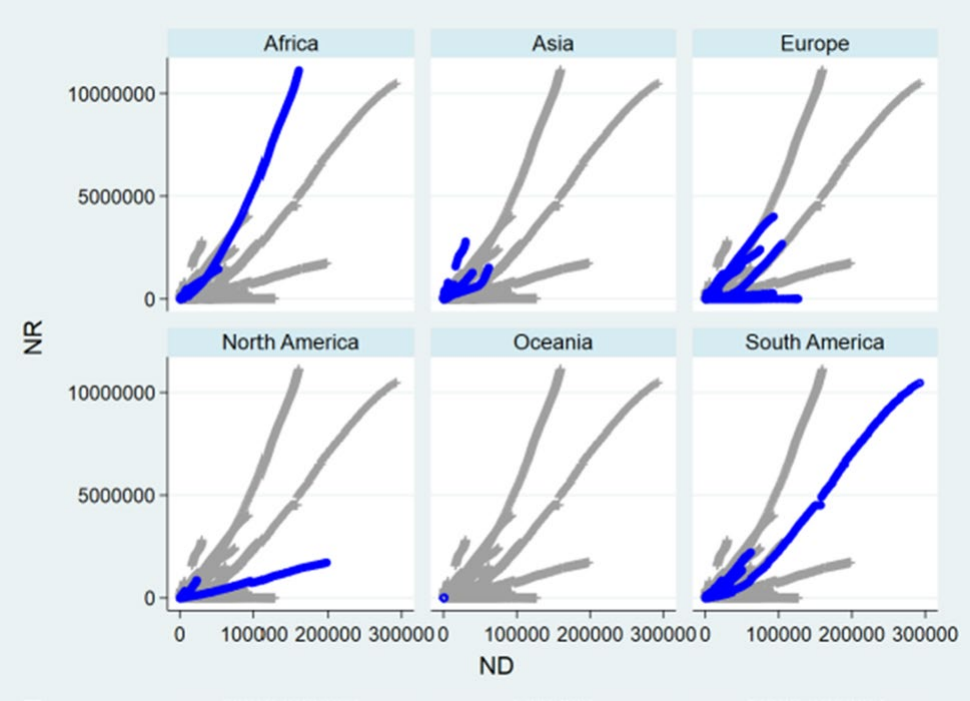
Relationship between ND and NR regarding the World

Before designing statistical strategies, the relationships between NC and ND, between NH and NC, and between NH and ND regarding China, the relationship between ND and NC regarding USA, and the relationships between NC and ND, between NC and NR, and between ND and NR regarding the World were depicted by front-and-back plots in Figs. 1, 2, 3, 4, 5, 6 and 7 [35]. Due to sparse distribution in Figs. 1, 2, 3 and asymptotic normality in Figs. 4, 5, 6 and 7, several linear and nonlinear panel regression models were considered as potential analytical methods when normality assumptions were violated.

### Tentative Analyses

Tentative analysis on the relationships between ND, NH, NR, and NC was performed by a one-stop solution for robust inference with multiway clustering (Stata package vcemway) [36]. In the sample, the identification code and day were identified as the clustered variables of interest. Thus, this study extended the ordinary least squares regression to incorporate random effects at the individual level. The following analyses estimated the resulting random effects model and adjusted its standard errors for two-way clustering in identification code and day. As compared with the statistical outcomes from the ordinary least squares regression, two-way clustering can lead to more conservative inferences than one-way clustering approaches.

### Panel Analyses

The main associations of interest in this study were panel associations of ND and NH with NC regarding China, panel associations between ND and NC regarding the USA, and panel associations of ND and NR with NC regarding the World. In the pooled regression analysis, the regions of China (Central China, Western China, Northeast China, and East China), the USA (New England, Mid-Atlantic Region, the South Region, Mid-West Region, the Southwest Region, and the West Region), and the World (Africa, Asia, Europe, North America, Oceania, and South America) were also considered as covariates.

The count data of ND, NH, NR, and NC tended to follow the Poisson or negative binomial distributions. In this large sample, the distributions approached to normal distributions approximately. Regarding the associations, the feasible panel models could be linear and nonlinear models. When NR, NH, NC, and ND were considered as count data, random-effects negative binomial regression and random-effects Poisson regression could be employed to reflect the associations of interest in nonlinear models. When ND, NH, NR, and NC were considered as continuous variables, pooled regression, stacked within-transformed linear regression, and quantile regression for panel data could be employed to explore the associations of interest in linear models.

Regarding China, the panel associations of ND and NH with NC could be found by using the regression model (1):

1 $$ {\text{NC}} \sim \beta_{0} + \beta_{{1}} {\text{NH }} + \beta_{{2}} {\text{ND}} + \mu_{{1}} . $$

Regarding the USA, the panel associations between ND and NC could be found by using the regression model (2):

2 $$ {\text{NC}} \sim \beta_{0} + \beta_{{1}} {\text{ND }} + \mu_{{2}} . $$

Regarding the World, the panel associations of ND and NR with NC could be found by using the regression model (3):

3 $$ {\text{NC}} \sim \beta_{0} + \beta_{{1}} {\text{NR }} + \beta_{{2}} {\text{ND}} + \mu_{{3}} . $$

Here, *β*_0_ was constant. *β*_1_ and *β*_2_ were coefficients. *μ*_1_, *μ*_2_, and *μ*_3_ were random errors. If optimized iterations were not concave, the possible calculations of chosen methods were deleted.

Regarding cubic or quadratic equations, this study aimed to explore the associations of interest rather than dynamic system analysis. Thus, it was unnecessary to conduct regressions with squared terms or interactions.

Pooled regressions are usually carried out to analyze available time series of cross-sections. The main advantage of pooled regression is the ability to measure different factors at the region level and aggregate results at the national level. The main disadvantages of pooled regression are overestimating and underestimating the impact in the regions.

Stacked within-transformed linear regression analysis was performed by Stata program xtstackreg [37]. Regarding the suitability and applicability, stacked within-transformed linear regression accommodated fixed-effects estimation, applied a degrees-of-freedom adjustment, and allowed for factor-variables in dependent variables. When regressing regarding China, the USA, and the World, all region-level units entered into regressions. After regression calculation, parts of the geographical covariates were left in the regression outcomes. The main advantage of stacked within-transformed linear regression is the ability to generate predictions from a “stacked” ensemble of models, including LASSO regression, k-nearest neighbors, random forest, and gradient boosting. This technique produces superior estimates with larger samples.

Quantile regression for panel data was performed by Stata program qregpd with Nelder–Mead optimization [38]. Likewise, quantile regression for panel data addresses a fundamental problem posed by alternative fixed-effect quantile estimators: inclusion of individual fixed effects alters the interpretation of the estimated coefficient on the treatment variable. Compared to the standard mean regression models, quantile regression models are more robust and flexible, which can help to account for unobserved heterogeneity and heterogeneous covariates effects. According to Powell (2015), a quantile regression estimator can be used to evaluate impacts of exogenous and endogenous treatment variables on an outcome distribution among the sample with small *T* [39]. Simultaneously, random-effects negative binomial regression and random-effects Poisson regressions were conducted.

### Panel Event Study

This study included panel models for the associations of interest and prediction models for the effects of key events. A panel event study implemented by the program “eventdd” in Stata [40] was employed to analyze how the key events influenced NC. With a difference-in-difference style model, a series of lag and lead coefficients and confidence intervals (CIs) were estimated and plotted. In the context, three key events were adopted as treatments regarding China, the USA, and the World (outside Antarctica, China, the USA, and MS Zaandam), respectively. On February 5, 2020, China released tax exemption and loan policies to beef up coronavirus containment (http://en.nhc.gov.cn/2020-02/06/c_76511.htm). Coronavirus Guidelines for America was issued on March 16, 2020 in the USA (https://www.whitehouse.gov/briefings-statements/coronavirus-guidelines-america/). On March 11, 2020, WHO characterized COVID-19 as a pandemic (https://www.who.int/emergencies/diseases/novel-coronavirus-2019/events-as-they-happen).

All analyses were performed with Stata (Version 14 and 16, Stata Corporation, College Station, TX, USA).

## Results

### Descriptive Analyses

Table 1 showed descriptive statistics for number of ND, NR, NH, and NC. Overall, there were 11,346 observations, 22,491 observations, and 81,408 observations of COVID-19 cases included during 366-day regarding China, 441-day regarding the USA, and 424-day regarding the World, respectively. Thus, the mean values of ND/NC, NR/NC, NH/NC, and NR/ND could reflect the control efficiency of COVID-19 pandemic. The mean values of ND/NC ratio regarding China, the USA, and the World were 0.032 (standard deviation (SD) = 0.416), 0.025 (SD = 0.019), and 0.026 (SD = 0.035), respectively. The mean value of NH/NC ratio regarding China were 2.975 (SD = 28.501), while mean value of NR/NC ratio regarding the World was 0.630 (SD = 0.320). The mean value of NH/ND ratio regarding China was 92.533(SD = 190.425), while mean value of NR/ND ratio regarding the World was 60.435 (SD = 151.284).

**Table 1:** Descriptive statistics of COVID-19ND, NC, and NR/NH cases

	Status	Days	Observations	Groups	Mean	Standard Deviation	Minimum	Maximum
China Provinces	NC	366	11,346	31	7.751	1292.399	− 68,149	68,149
	ND	366	11,346	31	0.408	85.725	− 4512	4512
	NH	366	11,346	31	7.257	1201.593	− 63,633	63,635
The USA States	NC	441	22,491	51	190,167.5	377,173.4	0	3,682,946
	ND	441	22,491	51	4053.384	7410.224	0	59,761
The World Countries	NR	424	81,408	192	151,272	694,941.2	0	1.20e+07
	NC	424	81,408	192	4003.126	16,491.61	0	292,752
	ND	424	81,408	192	110,795.8	604,092.7	0	1.11e+07

### Tentative Analyses

In Table 2, NC was significantly predicted by ND and NH regarding China. Simultaneously, NC was significantly predicted by ND regarding the USA. NC was significantly predicted by ND and NR regarding the World.

**Table 2:** Robust inference on NC coefficients (standardized errors)

	China	The USA	The World
ND	4.327*** (0.460)	55.359*** (5.836)	16.847** (7.163)
NR			0.725 *** (0.148)
NH	0.761*** (0.034)		
Constant	0.459 (0.938)	− 34,222.72 (44,680.19)	3465.593 (11,353.570)
*σ*_u_	0	121,713.44	88,327.022
*σ*_e_	166.948	109,692.49	130,359.990
*ρ*	0	0.552	0.315
*R* square			
Within	0.9833	0.8730	0.9330
Between	0.9999	0.7011	0.9635
Overall	0.9834	0.7735	0.9474
Groups	31	51	189
*N*	11,346	22,491	80,136

*N* number of observations

*p < 0.10, **p < 0.05, and ***p < 0.01

### Pooled Analyses

In Table 3, several important findings were obtained. In China, NH (coefficient = 0.761, 95% confidence interval (CI): 0.701, 0.821; *p* < 0.001), ND (coefficient = 4.327, 95% CI 3.503, 5.151; *p* < 0.001), Central China (coefficient = 0.540, 95% CI 0.403, 0.677; *p* < 0.001), East China (coefficient = 0.734, 95% CI 0.392, 1.076; *p* < 0.001), Northeast China (coefficient = 0.406, 95% CI 0.044, 0.768; *p* = 0.029), and Western China (coefficient = 0.204, 95% CI 0.081, 0.326; *p* = 0.002) had significantly positive associations with NC.

**Table 3:** Pooled regressions on NC regarding China, the USA, and the World, coefficients (standardized errors)

	China	The USA	The World
NH	0.761*** (0.029)		
NR			0.775*** (0.122)
ND	4.327*** (0.403)	46.696*** (7.651)	14.537*** (5.510)
Central China	0.540*** (0.067)		
East China	0.734*** (0.167)		
Northeast China	0.406** (0.177)		
Western China	0.204*** (0.060)		
Mid-West Region		20,342.730 (29,289.890)	
Mid-Atlantic Region		− 173,041.900** (78,755.480)	
New England		− 48,221.420 (33,839.030)	
South Region		39,905.910 (31,684.070)	
Southwest Region		66,525.850 (51,955.920)	
West Region		55,697.100** (25,556.030)	
Africa			5708.15* (3286.4)
Asia			5597.012 (5547.308)
Europe			45,411.11** (19,388.16)
North America			− 30,936.51 (22,541.11)
Oceania			44.037 (135.633)
South America			− 51,708.54 (38,046.72)
*R* square	0.9834	0.8508	0.9520
*N*	11,346	22,491	80,136

*N* number of observations

*p < 0.10, **p < 0.05, ***p < 0.01

In the USA, ND (coefficient = 46.696, 95% CI 31.329, 62.06335; *p* < 0.001) and the West Region (coefficient = 55,697.1, 95% CI 4366.306, 107,027.9; *p* = 0.034) had significantly positive associations with NC, while Mid-Atlantic Region (coefficient = − 173,041.9, 95% CI − 331,226.9, − 14,856.86; *p* = 0.033) had significantly negative association with NC.

Regarding the World, NR (coefficient = 0.775, 95% CI 0.534, 1.016; *p* < 0.001), ND (coefficient = 14.537, 95% CI 3.668, 25.407; *p* = 0.009), Africa (coefficient = 5708.15, 95% CI − 774.809, 12,191.11; *p* = 0.084), and Europe (coefficient = 45,411.11, 95% CI 7164.803, 83,657.41; *p* = 0.020) had significantly positive associations with NC.

### Panel Regressions

Before conducting random-effects Poisson regression and random-effects negative binomial regression, the 66 values of NC (< 0) were treated as missing values. The results from the estimation presented in Table 4 indicated that ND and regions had significant effects on NC regarding China.

**Table 4:** Regressions on NC in China’s province-level sample

	Stacked within-transformed linear regression	Random-effects Poisson regression	Random-effects negative binomial regression
	Coefficient (SE)	IRR (95% CI)	IRR (95% CI)
NH	0.761*** (0.029)	0.9999801 (0.9998661, 1.000094)	1.001162*** (1.00097, 1.001353)
ND	4.327*** (0.403)	1.001599* (0.9999862, 1.003215)	0.98516*** (0.9825326, 0.9877943)
Central China		36.46152*** (9.09499, 146.1731)	0.0302182*** (0.0269784, 0.033847)
East China		7.014822*** (4.82188, 10.20509)	0.104624*** (0.0981832, 0.1114873)
Northeast China		4.020119*** (1.739002, 9.293469)	0.065412*** (0.0565445, 0.07567)
Western China		2.578395*** (1.526414, 4.355385)	0.0511991*** (0.0468615, 0.0559382)
/lnalpha		0.0455876 (− 174.0498, 174.141)	
Alpha		1.046643 (2.58e−76, 4.25e+75)	
/ln_*r*			− 0.7272596 (− 1.188863, − 0.2656564)
/ln_*s*			2.078229 (1.257295, 2.899163)
*r*			0.4832314 (0.3045675, 0.7667025)
*s*			7.990303 (3.515897, 18.15893)
Western China	1.187*** (0.124)		
*N*	11,346	11,280	11,280

*SE* standardized errors, *IRR* incidence rate ratio, *95% CI* 95% confidence interval, *N* number of observations

*p < 0.10, **p < 0.05, ***p < 0.01

The results from the estimation presented in Table 5 indicated that ND had significant effects on NC in stacked within-transformed linear regression, quantile regression for panel data, random-effects Poisson regression, and random-effects negative binomial regression regarding the USA. Moreover, regions had significant effects on NC in random-effects Poisson regression regarding the USA.

**Table 5:** Panel regressions on NC in the sample of the USA (*N* = 22,491)

	Stacked within-transformed linear regression		Quantile regression for panel data		Random-effects Poisson regression		Random-effects negative binomial regression	
	Coefficient	95% CI	Coefficient	95% CI	IRR	95% CI	IRR	95% CI
ND	55.399***	43.748, 67.049	52.182*	44.638, 59.726	1.000078***	1.000044, 1.000113	1.000071***	1.000071, 1.000072
Mid-West Region	− 146,132.8	− 346,832.2, 54,566.49			115,936.5***	86,205.9, 155,920.6		
Mid-Atlantic Region					70,616.88***	41,226.98, 120,958.3		
New England					40,201.06***	20,368.76, 79,343.31		
South Region					132,256.6***	98,248.92, 178,035.6		
Southwest Region					112,052.2***	64,793.88, 193,778.9		
West Region					59,030.17***	34,186.97, 101,926.6		
/lnalpha	11.44237	− 58.80172, 81.68646			− 1.00736	− 159.3829, 157.3682		
Alpha	93,187.37	2.90e−26, 2.99e+35			0.3651817	6.04e−70, 2.21e+68		
/ln_*r*	0.0841999	− 0.2618117, 0.4302116						
/ln_*s*	10.7737	10.33666, 11.21074						
*r*	1.087846	0.7696559, 1.537583						
*s*	47,748.22	30,842.7, 73,919.99						

*NB regression* negative binomial regression, *IRR* incidence rate ratio, *95% CI* 95% confidence interval, *N* number of observations

*p < 0.10, **p < 0.05, ***p < 0.01

The results from the estimation presented in Table 6 indicated that ND and NR had significant effects on NC in stacked within-transformed linear regression and random-effects Poisson regression regarding the World. Moreover, regions had significant effects on NC in random-effects Poisson regression regarding the World.

**Table 6:** Regression on NC in the World sample

	Stacked within-transformed linear regression		Random-effects Poisson regression	
	Coefficient	95% CI	IRR	95% CI
ND	16.87534**	2.663998, 31.08668	1.000027***	1.000013, 1.00004
NR	0.7248077***	0.4321259, 1.01749	0.9999999**	0.9999997, 1.00000
Africa	11,725.53	− 81,099.9, 104,551	35,024.1***	14,553.94, 84,285.63
Asia			87,631.48***	59,470.78, 129,126.9
Europe			121,250.7***	81,578, 180,217
North America			31,700.67***	16,041.34, 62,646.44
Oceania			2132.171***	414.2913, 10,973.32
South America			154,141.3***	83,733.69, 283,751.1
/lnalpha			0.9214848	− 68.96149, 70.80446
Alpha			2.513019	1.12e−30, 5.62e+30
*N*	80,136		80,136	

*IRR* incidence rate ratio, *95% CI* 95% confidence interval, *N* number of observations

*p < 0.10, **p < 0.05, ***p < 0.01

### Panel Event Study

**Fig. 8:**
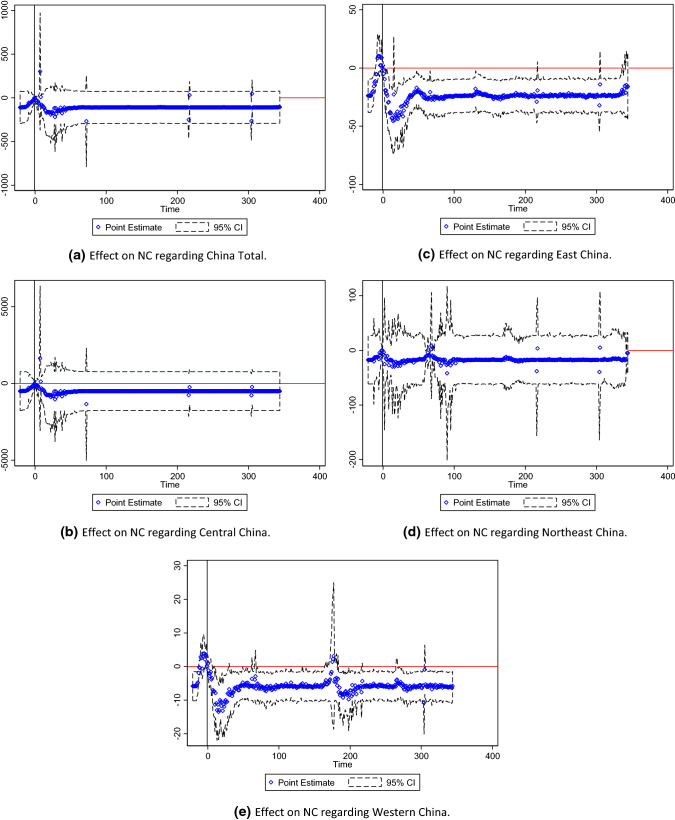
**a** Effect on NC regarding China total. **b** Effect on NC regarding Central China. **c** Effect on NC regarding East China. **d** Effect on NC regarding Northeast China. **e** Effect on NC regarding Western China

**Fig. 9:**
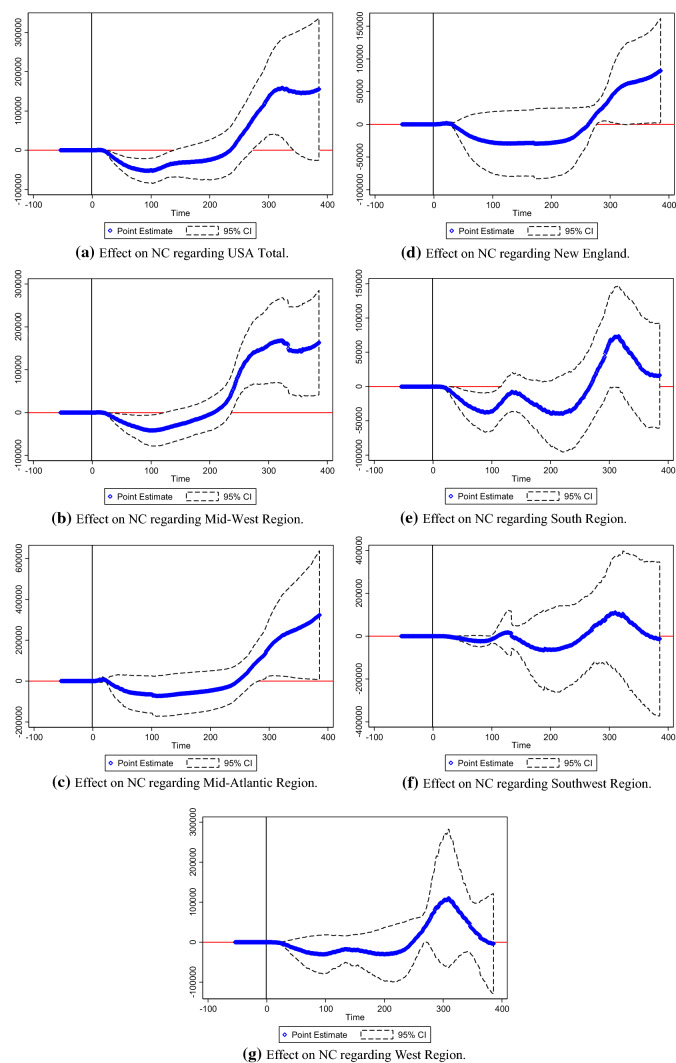
**a** Effect on NC regarding USA total. **b** Effect on NC regarding Mid-West Region. **c** Effect on NC regarding Mid-Atlantic Region. **d** Effect on NC regarding New England. **e** Effect on NC regarding South Region. **f** Effect on NC regarding Southwest Region. **g** Effect on NC regarding West Region

**Fig. 10:**
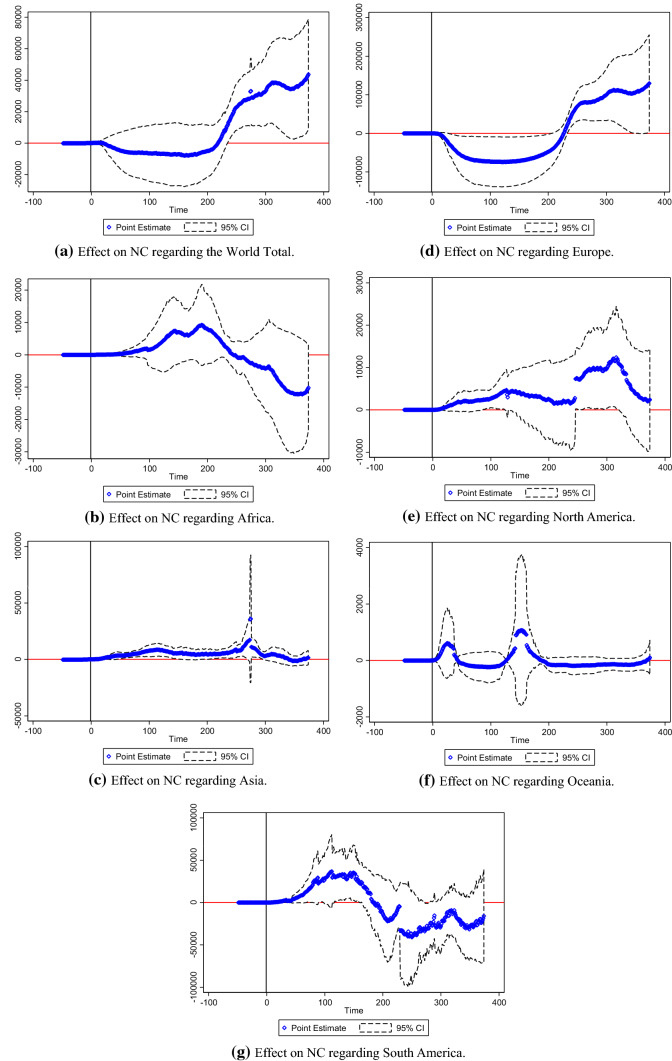
**a** Effect on NC regarding the World total. **b** Effect on NC regarding Africa. **c** Effect on NC regarding Asia. **d** Effect on NC regarding Europe. **e** Effect on NC regarding North America. **f** Effect on NC regarding Oceania. **g** Effect on NC regarding South America

Figure 8a–e reported NC trend following the key event regarding China Total, Central China, East China, Northeast China, and Western China. Figure 9a–g reported NC trend following the key event regarding the USA Total, Mid-West Region, Mid-Atlantic Region, New England, the South Region, the Southwest Region, and the West Region. Figure 10a–g reported NC trend following the key event regarding the World Total, Africa, Asia, Europe, North America, Oceania, and South America. Notably, point estimation curves regarding China were nearly straight, while the curves of point estimations and 95% CIs regarding the USA and the World were choppy and changeable with wave crests. Simultaneously, the differences between upper and lower limits regarding China approached to constants in the gross, while the differences between upper and lower limits regarding the USA and the World were changeable.

*R* squares regarding China Total, Central China, East China, Northeast China, and Western China were 0.9841, 0.9864, 0.9933, 0.9917, and 0.9895, respectively. *R* squares regarding the USA Total, Mid-West Region, Mid-Atlantic Region, New England, the South Region, the Southwest Region, and the West Region were 0.8056, 0.9259, 0.8886, 0.9131, 0.9654, 0.9899, and 0.9739, respectively. *R* squares regarding the World Total, Africa, Asia, Europe, North America, Oceania, and South America were 0.9488, 0.9984, 0.9934, 0.8921, 0.9958, 0.9861, and 0.9992, respectively.

Exponential rise of NC was plotted in the Figs. 9a–g and 10a, d. In the figures, the dates of turning points of NC were depicted. Thus, this study was effective to reflect effects of key events on NC of the COVID-19.

## Discussion

### Main Outcomes

This study employed publicly available daily datasets including the samples of China, the USA, and the World (outside Antarctica, China, the USA, and MS Zaandam) and obtained the associations of ND, NR, and NH with NC regarding China, the USA, and the World, respectively. In panel event study, curve lines showed key events influenced NC regarding the USA and the World significantly, while straight line showed key events nearly had no significant influence on NC regarding China.

Congruent with a prior study [41], this study confirmed the effects of control measures. Regression outcomes provided coarse estimates of controlling performance comparisons of COVID-19 pandemic. This study was in line with early simulation outcomes which found that their NH rates were the approximately linear increasing functions and the ND rates were the small constants [42]. This could partially explained by an early study which indicated that socio-economic determinants and city sizes had high impacts on the change of COVID-19 transmission in China [43]. Because of mean value of NH/NC ratio (China) > mean value of NR/NC ratio (the World) and mean value of NH/ND ratio (China) > mean value of NR/ND ratio (the World), the practical performance of COVID-19 controlling in China was seemly better than that in the other countries. Some Chinese scholars agreed with this judgment [44, 45].

With regard to methodologies, the findings in panel event study were in line with prior studies. For example, an exploratory data analysis with visualizations had been made to understand the number of NR, NC, and ND in China [46]. An 82-day (January 21 to April 12, 2020) forecast infections for COVID-19 death indicated that forecast placed the COVID-19 peak in the USA around July 14, 2020 [47]. This study was in line with another study which revealed that the effect of NC on ND was heterogeneous across provinces in China [48]. Furthermore, the spread of COVID-19 up to February 5, 2020 the number of NC showed a trend of “rapid increase before slowing down” [49]. Another forecast showed that the cumulative number of cases for Italy, UK and the USA corresponded to the diminishing average daily rate, from April 22 to May 22, 2020 [50].

Changes of COVID-19 ND, NH, NR, and NC in various regions could be influenced by life style, environmental factors, regulations, and progressing stages. Regarding life style, change in social distancing [51], increase of space–time clusters [52], and different sets of neighborhood characteristics [53] could be identified as risk factors for ND and NC during the COVID-19 pandemic. As to environmental factors, a study indicated temperature and the columnar density of total atmospheric ozone had a strong association with the tendency of COVID-19 spreading in almost all states in the USA [54]. As for regulations mainly including mobility restrictions and other non-pharmacological interventions, ill-prepared work [55], facemask shortage [56], poor traveller screening [57], forgone care [58], and population migration [59] could lead to ineffective prevention and controlling COVID-19. Regarding progressing stages, changes of COVID-19 ND, NH, NR, and NC might be caused by COVID-19 epidemic progressing laws differentially in various countries. Theoretically, various phases of COVID-19 epidemic documented four phases in 61 most affected countries [60], three or four phases in Wuhan City, Hubei Province and China [61], and five stages in China's non-Hubei provinces [62].

There were small curves in the point estimation regarding China and wide range of trajectories regarding the USA. This could be partially explained by several studies. For example, a study showed rapid nucleation and diffusion in January 2020 followed by rapid NC decrease in February in China, while the USA showed a wide range of trajectories, with an abrupt transition from slow NC increase in January and February, to rapid geographic dispersion shortly before mobility reductions occurred in March [63]. Regarding the epidemic trends of national and state regional administrative units, a study from July 27, 2020, to January 22, 2021 indicated the turning point of the early epidemic in the USA was predicted to occur in September [64]. Another model inferred that the inflection point of the epidemic across China would be mid-February, and the end of the epidemic would be in late March [65].

### Strengths and Weaknesses

Regarding data sources, this study employed three datasets. The current study had a large sample size which increased the precision of the study. Additionally, more than 1-year period could provide reliable results regarding epidemic control and daily changes in the prevalence of COVID-19 conditions. Regarding statistical methods, this study adopted several advanced panel regression methods. Especially, the event study with difference in difference was used to analyze the role of key events. Compared with the other studies [66–69], the results from this study were significantly more accurate, realistic, appropriate, and suitable for long-time series outbreak data. Another advantage of this study was under the consideration of key events.

There were several limitations. First, several variables including demographics, financial support, and international aids were not taken into account. Statistically, a study in South Korea found that sex, region, and infection reasons affected on both NR and ND [13]. Second, this study did not adopt newly designed methods conceived by the author to analyze the law of spread and transmission of COVID-19. Changes in case definitions affected inferences on the transmission dynamics of COVID-19 allowed detection of more cases as knowledge increased in China [70]. Finally, this study did consider one key event rather than varying treatment time and duration [71].

## Conclusion

Using panel analysis and data collected in China province-level units, the USA state-level units, and the World country-level units (outside Antarctica, China, the USA, and MS Zaandam), regressions confirmed the positive panel associations between NH, ND, and NC regarding China, between ND and NC regarding the USA, between NR, ND, and NC regarding the World. Panel event study showed key events influenced NC regarding the World and the USA more forceful and unsteady as compared to that regarding China. Future work on the basis of the current study should be performed on the influencing mechanism of the panel associations.

## Data Availability

The data that support the findings of this study are openly available in Harvard Dataverse at China Data Lab, 2020 [Titles: China COVID-19 Daily Cases with Basemap, US COVID-19 Daily Cases with Basemap, and World COVID-19 Daily Cases with Basemap].

## Appendix: Geographical Divisions

The main five regions in China are Central China (Hubei, Shaanxi, Anhui, Jiangxi, Henan, and Hunan), Western China (Neimenggu, Guangxi, Chongqing, Sichuan, Guizhou, Yunan, Xizang, Shanxi, Gansu, Qinghai, Ningxia, and Xinjiang), Northeast China (Liaoning, Jilin, and Heilongjiang), and East China (Beijing, Tianjin, Hebei, Shanghai, Jiangsu, Zhejiang, Fujian, Shandong, Guangdong, and Hainan).

According to official definition, the USA was divided into six regions: New England (Connecticut, Maine, Massachusetts, New Hampshire, Rhode Island, and Vermont), Mid–Atlantic Region (Delaware, Maryland, New Jersey, New York, Pennsylvania, and Washington D.C.), The South Region (Alabama, Arkansas, Florida, Georgia, Kentucky, Louisiana, Mississippi, North Carolina, South Carolina, Tennessee, Virginia, and West Virginia), Mid-West Region (Illinois, Indiana, Iowa, Kansas, Michigan, Minnesota, Missouri, Nebraska, North Dakota, Ohio, South Dakota, and Wisconsin), The Southwest Region (Arizona, New Mexico, Oklahoma, and Texas), and The West Region (Alaska, Colorado, California, Hawaii, Idaho, Montana, Nevada, Oregon, Utah, and Wyoming).

The World (outside Antarctica, China, the USA, and MS Zaandam) includes Africa (Algeria, Angola, Benin, Botswana, Burkina Faso, Burundi, Cabo Verde, Cameroon, Central African Republic, Chad, Comoros, Congo (Brazzaville), Congo (Kinshasa), Ivory Coast, Djibouti, Egypt, Equatorial Guinea, Eritrea, Eswatini, Ethiopia, Gabon, Gambia, Ghana, Guinea, Guinea-Bissau, Kenya, Lesotho, Liberia, Libya, Madagascar, Malawi, Mali, Mauritania, Mauritius, Morocco, Mozambique, Namibia, Niger, Nigeria, Rwanda, Sao Tome and Principe, Senegal, Seychelles, Sierra Leone, Somalia, South Africa, South Sudan, Sudan, Tanzania, Togo, Tunisia, Uganda, Zambia, and Zimbabwe), Asia (Afghanistan, Armenia, Azerbaijan, Bahrain, Bangladesh, Bhutan, Brunei, Burma, Cambodia, Cyprus, Diamond Princess, Georgia, India, Indonesia, Iran, Iraq, Israel, Japan, Jordan, Kazakhstan, Korea, South, Kuwait, Kyrgyzstan, Laos, Lebanon, Malaysia, Maldives, Mongolia, Nepal, Oman, Pakistan, Philippines, Qatar, Saudi Arabia, Singapore, Sri Lanka, Syria, Taiwan, Tajikistan, Thailand, Timor-Leste, Turkey, United Arab Emirates, Uzbekistan, Vietnam, and Yemen), Europe (Albania, Andorra, Austria, Belarus, Belgium, Bosnia and Herzegovina, Bulgaria, Croatia, Czechia, Denmark, Estonia, Finland, France, Germany, Greece, Holy See, Hungary, Iceland, Ireland, Italy, Kosovo, Latvia, Liechtenstein, Lithuania, Luxembourg, Malta, Moldova, Monaco, Montenegro, Netherlands, North Macedonia, Norway, Poland, Portugal, Romania, Russia, San Marino, Serbia, Slovakia, Slovenia, Spain, Sweden, Switzerland, Ukraine, United Kingdom, and West Bank and Gaza), North America (Antigua and Barbuda, Bahamas, Barbados, Belize, Canada, Costa Rica, Cuba, Dominica, Dominican Republic, El Salvador, Grenada, Guatemala, Haiti, Honduras, Jamaica, Mexico, Nicaragua, Panama, Saint Kitts and Nevis, Saint Lucia, Saint Vincent and the Grenadines, and Trinidad and Tobago), Oceania (Australia, Fiji, Marshall Islands, Micronesia, New Zealand, Papua New Guinea, Samoa, Solomon Islands, Vanuatu), and South America (Argentina, Bolivia, Brazil, Chile, Colombia, Ecuador, Guyana, Paraguay, Peru, Suriname, Uruguay, and Venezuela).

## Abbreviations

COVID-19: Coronavirus disease 2019
ND: Newly dead cases
NC: Newly confirmed cases
NR: Newly recovered cases
NH: Newly healed cases
IRR: Incidence rate ratio
CI: Confidence interval
SD: Standard deviation

## References

[1] Wells CR, Sah P, Moghadas SM, Pandey A, Shoukat A, Wang Y, Wang Z, Meyers LA, Singer BH, Galvani AP (2020). Impact of international travel and border control measures on the global spread of the novel 2019 coronavirus outbreak. Proc Natl Acad Sci USA.

[2] Chinazzi M, Davis JT, Ajelli M, Gioannini C, Litvinova M, Merler S, Pastore A, Piontti Y, Mu K, Rossi L, Sun K, Viboud C, Xiong X, Yu H, Halloran ME, Longini IM, Vespignani A (2020). The effect of travel restrictions on the spread of the 2019 novel coronavirus (COVID-19) outbreak. Science.

[3] Neelon B, Mutiso F, Mueller NT, Pearce JL, Benjamin-Neelon SE (2021). Spatial and temporal trends in social vulnerability and COVID-19 incidence and death rates in the United States. PLoS ONE.

[4] Kim S, Seo YB, Jung E (2020). Prediction of COVID-19 transmission dynamics using a mathematical model considering behavior changes. Epidemiol Health.

[5] Rahimi F, Talebi Bezmin Abadi A (2020). Practical strategies against the novel coronavirus and COVID-19-the Imminent Global Threat. Arch Med Res.

[6] Ghanchi A (2020). Adaptation of the national plan for the prevention and fight against pandemic influenza to the 2020 COVID-19 epidemic in France. Disaster Med Public Health Prep.

[7] He J, Chen G, Jiang Y (2020). Comparative infection modeling and control of COVID-19 transmission patterns in China, South Korea, Italy and Iran. Sci Total Environ.

[8] Awan TM, Aslam F (2020). Prediction of daily COVID-19 cases in European countries using automatic ARIMA model. J Public Health Res.

[9] Anastassopoulou C, Russo L, Tsakris A, Siettos C (2020). Data-based analysis, modelling and forecasting of the COVID-19 outbreak. PLoS ONE.

[10] Wu JT, Leung K, Leung GM (2020). Nowcasting and forecasting the potential domestic and international spread of the 2019-nCoV outbreak originating in Wuhan, China: a modelling study. Lancet.

[11] Li M, Zhang Z, Jiang S, Liu Q, Chen C, Zhang Y, Wang X (2020). Predicting the epidemic trend of COVID-19 in China and across the world using the machine learning approach. medRxiv.

[12] Ji T, Chen HL, Xu J, Wu LN, Li JJ, Chen K, Qin G (2020). Lockdown contained the spread of 2019 novel coronavirus disease in Huangshi city, China: early epidemiological findings. Clin Infect Dis.

[13] Al-Rousan N, Al-Najjar H (2020). Data analysis of coronavirus COVID-19 epidemic in South Korea based on recovered and death cases. J Med Virol.

[14] Wood SN. Inferring UK COVID-19 fatal infection trajectories from daily mortality data: were infections already in decline before the UK lockdowns? [published online ahead of print, 2021 Mar 30]. Biometrics. 2021. 10.1111/biom.13462. 10.1111/biom.13462 PMC8251436 33783826

[15] Daniyal M, Ogundokun RO, Abid K, Khan MD, Ogundokun OE (2020). Predictive modeling of COVID-19 death cases in Pakistan. Infect Dis Model.

[16] Yadav RP, Verma R (2020). A numerical simulation of fractional order mathematical modeling of COVID-19 disease in case of Wuhan China. Chaos Solitons Fractals.

[17] Jin R (2021). The lag between daily reported Covid-19 cases and deaths and its relationship to age. J Public Health Res.

[18] Verma V, Vishwakarma RK, Verma A, Nath DC, Khan HTA (2020). Time-to-death approach in revealing chronicity and severity of COVID-19 across the world. PLoS ONE.

[19] Chowdhury R, Sneddon G, Hasan MT (2020). Analyzing the effect of duration on the daily new cases of COVID-19 infections and deaths using bivariate Poisson regression: a marginal conditional approach. Math Biosci Eng.

[20] Alberti T, Faranda D (2020). On the uncertainty of real-time predictions of epidemic growths: a COVID-19 case study for China and Italy. Commun Nonlinear Sci Numer Simul.

[21] Bartolomeo N, Trerotoli P, Serio G (2021). Short-term forecast in the early stage of the COVID-19 outbreak in Italy. Application of a weighted and cumulative average daily growth rate to an exponential decay model. Infect Dis Model.

[22] Saba T, Abunadi I, Shahzad MN, Khan AR (2021). Machine learning techniques to detect and forecast the daily total COVID-19 infected and deaths cases under different lockdown types [published online ahead of print, 2021 Feb 1]. Microsc Res Tech.

[23] YeŞİlkanat CM (2020). Spatio-temporal estimation of the daily cases of COVID-19 in worldwide using random forest machine learning algorithm. Chaos Solitons Fractals.

[24] Zhao H, Merchant NN, McNulty A (2021). COVID-19: short term prediction model using daily incidence data. PLoS ONE.

[25] Talkhi N, Akhavan Fatemi N, Ataei Z, Jabbari Nooghabi M (2021). Modeling and forecasting number of confirmed and death caused COVID-19 in IRAN: a comparison of time series forecasting methods. Biomed Signal Process Control.

[26] Shastri S, Singh K, Kumar S, Kour P, Mansotra V (2020). Time series forecasting of Covid-19 using deep learning models: India-USA comparative case study. Chaos Solitons Fractals.

[27] Palmer WR, Davis RA, Zheng T (2021). Count-valued time series models for COVID-19 daily death dynamics. Stat (Int Stat Inst)..

[28] Takefuji Y (2021). Fourier analysis using the number of COVID-19 daily deaths in the US. Epidemiol Infect.

[29] Smith BA (2020). A novel IDEA: the impact of serial interval on a modified-Incidence Decay and Exponential Adjustment (m-IDEA) model for projections of daily COVID-19 cases. Infect Dis Model.

[30] China Data Lab. 2020. Policies and regulations. 10.7910/DVN/OAM2JK. Harvard Dataverse, V9, UNF:6:JqS0gAZjLDn7OqaXP/ZvNw== [fileUNF]

[31] Gecili E, Ziady A, Szczesniak RD (2021). Forecasting COVID-19 confirmed cases, deaths and recoveries: revisiting established time series modeling through novel applications for the USA and Italy. PLoS ONE.

[32] China Data Lab. 2020. China COVID-19 daily cases with Basemap. 10.7910/DVN/MR5IJN. Harvard Dataverse, V18, UNF:6:6UaA0wJ4LY1Cv2AJJQkXRQ== [fileUNF]

[33] China Data Lab. 2020. US COVID-19 daily cases with Basemap. 10.7910/DVN/HIDLTK. Harvard Dataverse, V17, UNF:6:EoMk3PaaWyd/Fe78Vw8gQw== [fileUNF]

[34] China Data Lab. 2020. World COVID-19 daily cases with Basemap. 10.7910/DVN/L20LOT. Harvard Dataverse, V13, UNF:6:VD511Y14oh70r+RohASKnQ== [fileUNF]

[35] Cox NJ (2021). Speaking Stata: front-and-back plots to ease spaghetti and paella problems. Stata J.

[36] Gu A, Yoo HI (2019). vcemway: a one-stop solution for robust inference with multi-way clustering. Stata J.

[37] Tauchmann H, Oberfichtner M. STACKREG: stata module to perform stacked linear regression analysis to facilitate testing of multiple hypotheses. Statistical Software Components S458913, Boston College Department of Economics; 2021.

[38] Matthew Baker. QREGPD: stata module to perform quantile regression for panel data. Statistical Software Components S458157, Boston College Department of Economics; 2016.

[39] Powell D. Quantile regression with nonadditive fixed effects. RAND Labor and Population Working Paper. 2015.

[40] Clarke D, Tapia Schythe K. EVENTDD: stata module to panel event study models and generate event study plots. Statistical Software Components S458737, Boston College Department of Economics; 2020.

[41] Guo Q, He Z (2021). Prediction of the confirmed cases and deaths of global COVID-19 using artificial intelligence. Environ Sci Pollut Res Int.

[42] Pei L, Zhang M (2021). Long-term predictions of current confirmed and dead cases of COVID-19 in China by the non-autonomous delayed epidemic models [published online ahead of print, 2021 Jul 26]. Cogn Neurodyn.

[43] Yang XD, Su XY, Li HL, Ma RF, Qi FJ, Cao YE (2021). Impacts of socio-economic determinants, spatial distance and climate factors on the confirmed cases and deaths of COVID-19 in China. PLoS ONE.

[44] Xu M, Cao C, Zhang X (2021). Fine-scale space-time cluster detection of COVID-19 in Mainland China using retrospective analysis. Int J Environ Res Public Health.

[45] Zhang XS, Vynnycky E, Charlett A, De Angelis D, Chen Z, Liu W (2021). Transmission dynamics and control measures of COVID-19 outbreak in China: a modelling study. Sci Rep.

[46] Dey SK, Rahman MM, Siddiqi UR, Howlader A (2020). Analyzing the epidemiological outbreak of COVID-19: a visual exploratory data analysis approach. J Med Virol.

[47] Arias Velásquez RM, Mejía Lara JV (2020). Forecast and evaluation of COVID-19 spreading in USA with reduced-space Gaussian process regression. Chaos Solitons Fractals.

[48] Sarkodie SA, Owusu PA (2020). Investigating the cases of novel coronavirus disease (COVID-19) in China using dynamic statistical techniques. Heliyon.

[49] Shi J, Gao X, Xue S (2021). Spatio-temporal evolution and influencing mechanism of the COVID-19 epidemic in Shandong province, China. Sci Rep.

[50] Şahin U, Şahin T (2020). Forecasting the cumulative number of confirmed cases of COVID-19 in Italy, UK and USA using fractional nonlinear grey Bernoulli model. Chaos Solitons Fractals.

[51] Amiri A (2021). Role of social distancing in tackling COVID-19 during the first wave of pandemic in Nordic region: evidence from daily deaths, infections and needed hospital resources. Int J Nurs Sci.

[52] Hohl A, Delmelle EM, Desjardins MR, Lan Y (2020). Daily surveillance of COVID-19 using the prospective space–time scan statistic in the United States. Spat Spatiotemporal Epidemiol.

[53] Kim B, Rundle AG, Goodwin ATS (2021). COVID-19 testing, case, and death rates and spatial socio-demographics in New York City: an ecological analysis as of June 2020. Health Place.

[54] Rui R, Tian M, Tang ML, Ho GT, Wu CH (2021). Analysis of the spread of COVID-19 in the USA with a spatio-temporal multivariate time series model. Int J Environ Res Public Health.

[55] Gilbert M, Pullano G, Pinotti F, Valdano E, Poletto C, Boëlle PY, D'Ortenzio E, Yazdanpanah Y, Eholie SP, Altmann M, Gutierrez B, Kraemer MUG, Colizza V (2020). Preparedness and vulnerability of African countries against importations of COVID-19: a modelling study. Lancet.

[56] Wu HL, Huang J, Zhang CJP, He Z, Ming WK (2020). Facemask shortage and the novel coronavirus disease (COVID-19) outbreak: reflections on public health measures. EClinicalMedicine.

[57] Gostic K, Gomez AC, Mummah RO, Kucharski AJ, Lloyd-Smith JO (2020). Estimated effectiveness of symptom and risk screening to prevent the spread of COVID-19. Elife.

[58] Anderson KE, McGinty EE, Presskreischer R, Barry CL (2021). Reports of forgone medical care among US adults during the initial phase of the COVID-19 pandemic. JAMA Netw Open.

[59] Hu Z, Wu Y, Su M (2021). Population migration, spread of COVID-19, and epidemic prevention and control: empirical evidence from China. BMC Public Health.

[60] Long C, Fu XM, Fu ZF (2020). Global analysis of daily new COVID-19 cases reveals many static-phase countries including the United States potentially with unstoppable epidemic. World J Clin Cases.

[61] Zhang Y, Yu B, Chen X, Rich S, Mo Q, Yan H (2021). Dynamics of the coronavirus disease 2019 (COVID-19) epidemic in Wuhan City, Hubei Province and China: a second derivative analysis of the cumulative daily diagnosed cases during the first 85 days. Glob Health J.

[62] Chen Y, Li Q, Karimian H, Chen X, Li X (2021). Spatio-temporal distribution characteristics and influencing factors of COVID-19 in China. Sci Rep.

[63] Small C, Sousa D (2021). Spatiotemporal evolution of COVID-19 infection and detection within night light networks: comparative analysis of USA and China. Appl Netw Sci.

[64] Zheng Z, Xie Z, Qin Y, Wang K, Yu Y, Fu P (2021). Exploring the influence of human mobility factors and spread prediction on early COVID-19 in the USA. BMC Public Health.

[65] Shang C, Yang Y, Chen GY, Shang XD (2021). A simple transmission dynamics model for predicting the evolution of COVID-19 under control measures in China. Epidemiol Infect.

[66] Triacca M, Triacca U (2021). Forecasting the number of confirmed new cases of COVID-19 in Italy for the period from 19 May to 2 June 2020. Infect Dis Model.

[67] ArunKumar KE, Kalaga DV, Sai Kumar CM, Chilkoor G, Kawaji M, Brenza TM (2021). Forecasting the dynamics of cumulative COVID-19 cases (confirmed, recovered and deaths) for top-16 countries using statistical machine learning models: auto-regressive integrated moving average (ARIMA) and seasonal auto-regressive integrated moving average (SARIMA). Appl Soft Comput.

[68] Barría-Sandoval C, Ferreira G, Benz-Parra K, López-Flores P (2021). Prediction of confirmed cases of and deaths caused by COVID-19 in Chile through time series techniques: a comparative study. PLoS ONE.

[69] Shaharudin SM, Ismail S, Hassan NA, Tan ML, Sulaiman NAF (2021). Short-term forecasting of daily confirmed COVID-19 cases in Malaysia using RF-SSA model. Front Public Health.

[70] Tsang TK, Wu P, Lin Y, Lau EHY, Leung GM, Cowling BJ (2020). Effect of changing case definitions for COVID-19 on the epidemic curve and transmission parameters in mainland China: a modelling study. Lancet Public Health.

[71] Dettmann E, Giebler A, Weyh A. flexpaneldid: a stata command for causal analysis with varying treatment time and duration. IWH Discussion Papers 5/2019, Halle Institute for Economic Research (IWH); 2019.

